# Investigating the gut bacteria structure and function of hibernating bats through 16S rRNA high-throughput sequencing and culturomics

**DOI:** 10.1128/msystems.01463-24

**Published:** 2025-04-09

**Authors:** Jian Zhou, Ying Liu, Tao Gu, Jingzhu Zhou, Fengming Chen, Shijun Li

**Affiliations:** 1 School of Public Health, the Key Laboratory of Environmental Pollution Monitoring and Disease Control, Ministry of Education, Guizhou Medical University 74628, Guiyang, Guizhou, China; 2 Guizhou Center for Disease Control and Prevention, Guiyang, Key Laboratory of Microbio and Infectious Disease Prevention and Control in Guizhou Province, Guiyang, Guizhou, China; Chinese Academy of Sciences, Beijing, China

**Keywords:** hibernating bats, gut bacteria, high-throughput sequencing, culturomics, 16S rRNA

## Abstract

The gut microbiota of bats is vital for their roles in health and the ecosystem, yet studies on hibernating bats in southwest China, particularly in the unique karst landscape of Guizhou, are limited. We captured three hibernating bat species—Pipistrellus (PB), Rhinolophus (RB), and Myotis (MB)—in Liping County, collecting rectal samples for 16S rRNA amplicon sequencing. Data processing involved Trimmomatic, Flash, and Qiime2 for operational taxonomic unit (OTU) standardization and species annotation via the Greengenes database. Differential abundance was analyzed using LEfSe, and diversity metrics were assessed through alpha and beta diversity analyses. The RB group was predominantly composed of *Proteobacteria* (80.99%), while MB and PB exhibited diverse compositions with significant OTU richness (729 in MB). Notable genera included *Hafnia* and *Yersinia* in RB and *Cosenzaea myxofaciens* in MB. High proportions of unclassified taxa were observed, particularly in RB (83.81%). Functional predictions indicated metabolic pathways, with a significant representation of human diseases in PB. Culturomics revealed the successful cultivation of *Huaxiibacter chinensis* and *Enterobacter chengduensis* from bats for the first time and appears to have identified a new bacterium that is likely closely related to *Clostridium paraputrificum*.

**IMPORTANCE:**

Our research reveals significant differences in the composition and diversity of the gut microbiota among three bat groups (PB, MB, and RB) from Guizhou. While Proteobacteria predominates in all groups, its abundance varies. Notably, the high richness of operational taxonomic units (OTUs) in the MB group suggests a more diverse microbial ecosystem, underscoring the complex interactions between species diversity, diet, gut microbiota, and overall ecological dynamics in bats. Furthermore, the substantial presence of unknown bacterial species in their intestines highlights the critical importance of cultivation-based approaches. The presence of specific taxa may have potential health implications for both bats and humans. These findings emphasize the need for further investigations into the functional roles of these microbiota and their contributions to host health. Future research should focus on longitudinal studies to elucidate these intricate interactions.

## INTRODUCTION

Bats are among the most diverse groups of mammals globally, playing crucial ecological roles and maintaining ecosystem balance. Situated in the southwestern part of China, Guizhou Province features a unique karst landscape with an average elevation of 1,100 meters.

Recent studies have highlighted that the gut microbiota of bats significantly influences their health and physiological functions while also playing vital roles in ecosystem functionality, pathogen transmission, and human health (1, 2). During hibernation, bats exhibit markedly reduced metabolic activity, leading to significant changes in the composition and functionality of their gut microbiota. Understanding these shifts is essential for elucidating the ecological adaptation mechanisms of bats and the principles of microbial ecology (3, 4). Furthermore, the species diversity of gut microbiota may differ among various bat species (5, 6).

High-throughput sequencing technologies, particularly 16S rRNA gene sequencing, have emerged as pivotal tools for investigating microbial community composition and functionality. This method allows researchers to delve into the interactions between gut microbiota and their hosts by analyzing the microbial diversity and abundance (7–9). Compared to traditional culture-based techniques, high-throughput sequencing provides an efficient and comprehensive insight into the complex structures of microbial communities (10). However, the importance of culture-based approaches for isolating pathogenic microbes cannot be overlooked (11, 12).

Interest in bat gut microbiota research has surged, encompassing diverse bat species across different regions and examining their relationships with environmental factors and host health (4–6). Despite these advances, systematic studies focusing on the gut microbiota of hibernating bats, particularly in the specific context of southwestern China, remain scarce. This study aims to employ 16S high-throughput sequencing in conjunction with culturomics to systematically analyze the gut microbial composition and functional characteristics of various bat species during hibernation in this region. Our research not only addresses the regional gap in bat microbiota studies but also expands the understanding of bat–microbe interactions, offering potential insights for public health, particularly regarding bats as vectors of pathogens (13, 14).

## MATERIALS AND METHODS

### Sample collection and preparation

A total of 24 bats, comprising nine Pipistrellus bats (PB), nine Rhinolophus bats (RB), and six Myotis bats (MB), were captured during their hibernation period from two caves in Liping County, Qiandongnan, Guizhou Province. The two caves are geographically proximate 4 km apart with comparable altitudes, temperatures, and humidity levels (Table S1). Detailed biometric parameters (body weight and sex) and environmental metadata for all 24 bats are documented in Table S1. Upon capture, the bats were immediately transported to the laboratory, where they were dissected under sterile conditions. Live bats were anesthetized with ether vapor (exposed to a closed container for 10 minutes to confirm no pain reflexes) and promptly decapitated by experienced experimenters to ensure a rapid and painless process. This research complied with all relevant wildlife protection laws, ensuring the welfare of the captured animals. Approximately 200 mg of rectal tissue (containing feces) was excised using sterile scissors and placed into 2 mL sterile cryovials containing brain heart infusion (BHI) medium, with appropriate labeling. Each sample was then homogenized in a sterile environment. For high-throughput sequencing and analysis, 18 samples were selected, divided into three groups: PB (sample number: CD1-6), RB (sample number: CD7-12), and MB (sample number: CD13-18), with six samples in each group. The remaining three RB (sample number: samples 1–3) and three PB (sample number: samples 4–6) gut samples were designated for culturomics.

### DNA extraction and 16S amplicon sequencing

The rectal tissue (containing feces) DNA from the 18 bats was extracted following the standardized protocol provided by a bacterial DNA extraction kit (Hangzhou Baiju Technology Co., Ltd.). High-throughput sequencing of the 16S rRNA gene was conducted by Shenzhen Weikemeng Technology Group, and 16S rRNA genes of distinct regions (16S V3–V4) were amplified using specifically the 341F (5′-CCTAYGGGRBGCASCAG-3′) 806R primer (5′-GGACTACNNGGGTATCTAAT-3′) with the barcode. Sequencing libraries were generated using the NEB Next Ultra DNA Library Prep Kit (lllumina, USA) following the manufacturer’s recommendations, and index codes were added. The library quality was assessed on the Agilent 5400 (Agilent Technologies Co Ltd., USA). At last, the library was sequenced on an Illumina platform, and 250 bp paired-end reads were generated.

### Sequencing data processing

Raw sequence data were processed using Trimmomatic (version 0.35), employing a sliding window approach. Sequences with a quality score below 20 were trimmed, and sequences shorter than 50 bp were discarded. The remaining paired-end raw data were merged using Flash software, yielding complete paired-end sequences. Quality control, denoising, merging, and chimera removal of the original sequences were performed using the DADA2 plugin (V. 1.22.0) in Qiime2 (V. 2022.2) (15, 16). This process resulted in standardized OTUs.

### Culturomics and species identification

Culturomics was conducted on gut specimens from PB and RB. Key culturing conditions established by Lagier et al. included the use of sheep blood and rumen fluid as critical culture materials (17, 18). Accordingly, four essential culture media were set up: Columbia blood agar, BHI supplemented with 8% sheep blood, BHI with 8% rumen fluid, and BHI agar plates. Cultures were incubated at 28°C and 37°C under anaerobic (anaerobic bags), microaerophilic (microaerophilic bags), and aerobic conditions. PB and RB samples were plated on the aforementioned media, and after incubation periods of 48 and 72 hours, colonies were picked based on established protocols and sub-cultured for isolation and purification over three generations. Single purified colonies were first identified using matrix-assisted laser desorption/ionization time-of-flight mass spectrometry (MALDI-TOF MS), instrument and model: Autof MS 1000 (manufactured by Antu Bio Co., China), with an identification score exceeding 9 indicating species-level confidence (19, 20). For strains with scores below 9, DNA was extracted, and the 16S rRNA gene was amplified using primers 27F (5′-AGTTTGATCMTGGCTCAG-3′) and 1492R (5′-AGTTTGATCMTGGCTCAG-3′), followed by sequencing at Beijing Tianyi Huiyuan Biotechnology Co., Ltd. The obtained 16S rRNA sequences were compared against the BLAST of the NCBI database to confirm the identity of the strains. We followed a widely accepted criterion for defining new species, where 16S rRNA gene sequence similarity greater than 98.7% is considered indicative of the same species, while a similarity below 98.7% is regarded as suggestive of a potential new species (21, 22).

### Data analysis

OTUs were categorized into PB, MB, and RB groups, each consisting of six samples. OTUs were aligned against the Greengenes2 database (V. release 2022.10) to obtain taxonomic annotation, encompassing species-level classifications from kingdom to species (23). In the presentation of species annotation results, only the top 20 most abundant species within each group were displayed, with the remaining lower-abundance species classified as “Other.” The LEfSe method was employed to analyze differential abundance among groups based on relative abundance tables and presented by the linear discriminant analysis score (24). Alpha diversity and beta diversity analyses were primarily conducted using the Qiime2 diversity plugin (16). Alpha diversity was assessed through metrics including Chao1, Faith’s phylogenetic diversity (Faith-pd), observed features, and Shannon and Simpson indices, where higher values indicate greater species complexity. The Wilcoxon test was utilized to evaluate significant differences in alpha diversity indices among groups. Beta diversity analyses included non-metric multidimensional scaling (NMDS), principal coordinate analysis (PCoA), and principal component analysis (PCA). To compare microbial community structures across different samples, the Bray–Curtis, weighted UniFrac, and unweighted UniFrac distances were calculated based on OTU abundance information. To investigate the phylogenetic relationships of OTUs, one representative OTU of the highest abundance from each annotated genus was selected, and a phylogenetic tree of the top 50 genera was constructed using the ggtree package in R (25). To predict microbial community functions, the principles of PICRUSt2 were applied based on sequences from the KEGG and MetaCyc databases of previously sequenced microbial genomes (26). Functional annotations from the KEGG database were categorized into three hierarchical levels (L1, L2, and L3). All data analyses were completed using the Wekemo Bioincloud platform (https://www.bioincloud.tech) (27).

## RESULTS

### Species annotation and richness

**Fig 1:**
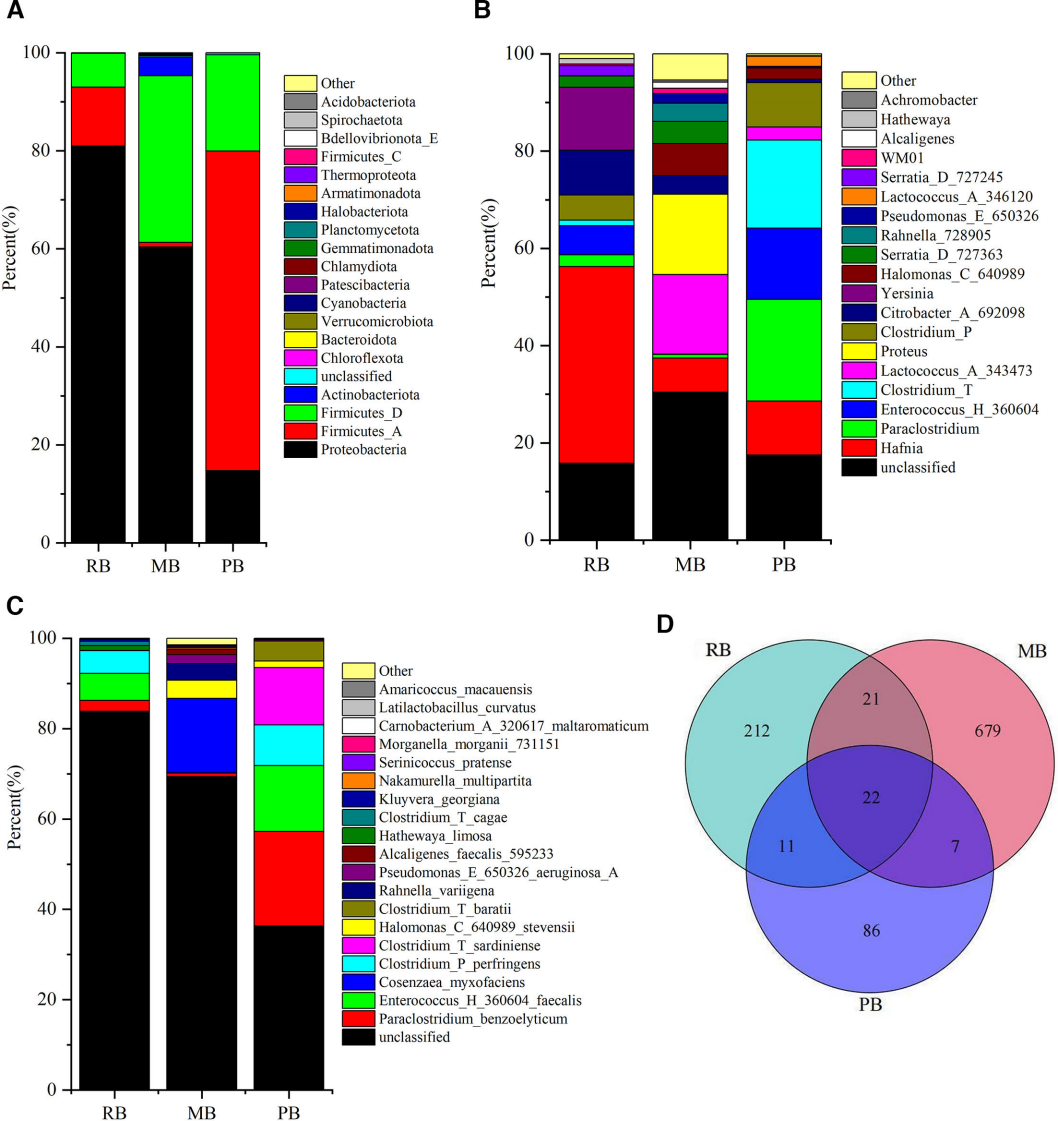
Relative distribution of bacterial species richness. (A) Bar chart depicting the relative distribution at the phylum level (top 20 by relative abundance). (B) Bar chart showing the relative distribution at the genus level (top 20 by relative abundance). (C) Bar chart illustrating the relative distribution at the species level (top 20 by relative abundance). (D) Venn diagram of OTUs.

The annotation results revealed a total of two microbial kingdoms: Bacteria and Archaea. We identified 28 phyla, 50 classes, 138 orders, 202 families, 382 genera, and 464 species. We compared the top 20 taxa at the phylum, genus, and species levels across the three experimental groups: RB, MB, and PB. At the phylum level, the RB group exhibited the highest annotation richness in *Proteobacteria* (80.99%), followed by *Firmicutes _A* (12.00%) and *Firmicutes_D* (6.89%). In contrast, the MB group showed a dominant presence of *Proteobacteria* (60.37%), Firmicutes_D (34.02%), and *Actinobacteriota* (3.76%). The PB group, meanwhile, was primarily annotated in *Firmicutes _A* (65.20%), *Firmicutes_D* (19.60%), and *Proteobacteria* (14.76%) (Fig. 1A). At the genus level, excluding the “unclassified” category, the RB group had the highest annotations in *Hafnia* (40.44%), *Yersinia* (12.91%), *Citrobacter_A*(692098) (9.23%), and *Enterococcus_H* (360604) (6.00%). The MB group predominantly annotated *Proteus* (16.50%), *Lactococcus_A* (343473) (16.35%), *Hafnia* (7.00%), and *Halomonas_C* (640989) (6.60%). For the PB group, the most annotated genera were *Paraclostridium* (20.91%), *Clostridium_T* (18.10%), *Enterococcus_H* (360604) (14.63%), and *Hafnia* (11.09%). Notably, the “unclassified” taxa accounted for a substantial proportion in all groups, particularly in the MB group (30.41%), followed by the RB group (15.82%) and the PB group (20.91%) (Fig. 1B).

At the species level, the RB group showed the highest annotations for *Enterococcus faecalis* (5.97%), *Clostridium perfringens* (5.05%), *Paraclostridium benzoelyticum* (2.41%), and *Hathewaya limosa* (1.09%). The MB group’s highest annotations were observed in *Cosenzaea myxofaciens* (16.50%), *Halomonas stevensii* (3.98%), *Rahnella variigena* (3.72%), and *Pseudomonas aeruginosa* (1.96%). In the PB group, the most annotated species included *Paraclostridium benzoelyticum* (20.91%), *Enterococcus faecalis* (14.57%), *Clostridium sardiniense* (12.70%), and *Clostridium perfringens* (8.97%). Additionally, a significant proportion of taxa remained “unclassified,” particularly in the RB group (83.81%), followed by the MB group (69.35%) and the PB group (36.35%) (Fig. 1C).

A Venn diagram illustrating the number of OTUs revealed that the MB group possessed the highest OTU count (729), followed by the RB group (266), with the PB group showing the least (126). Notably, only 22 OTUs were shared among the three groups (Fig. 1D).

**Fig 2:**
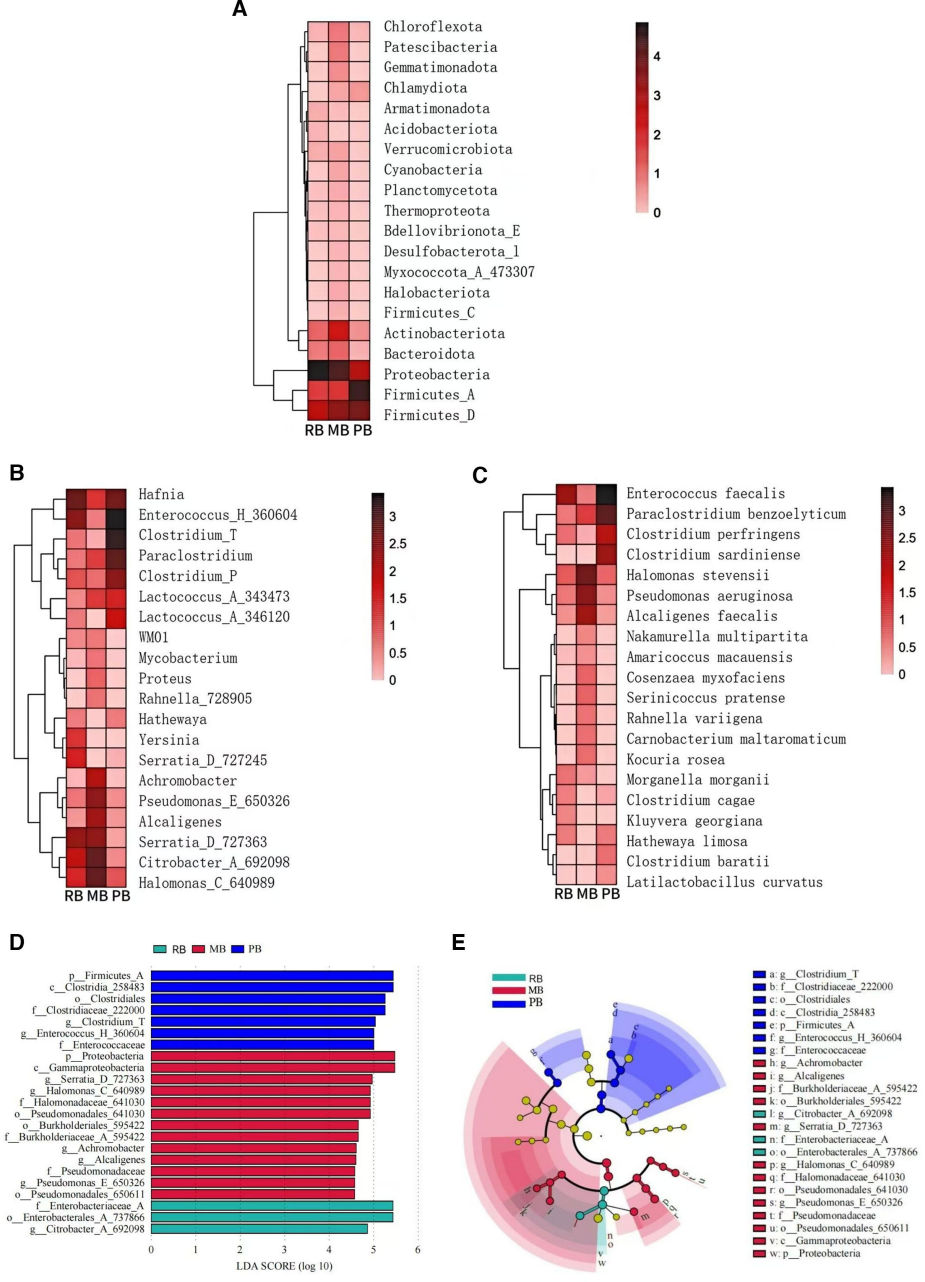
Clustering and LEfSe analysis. Hierarchical clustering heatmap displaying the absolute abundance of the top 20 taxa, with a clustering tree on the left illustrating the similarity in species abundance distributions across groups. The central heatmap represents the log10-transformed absolute abundance. (A) Clustering heatmap at the phylum level. (B) Clustering heatmap at the genus level. (C) Clustering heatmap at the species level. (D) LDA bar chart from LEfSe analysis at the genus and higher taxonomic levels, with each horizontal bar representing a species, where the length corresponds to the LDA score and longer bars indicate greater differences. (E) Cladogram from LEfSe analysis. The cladogram depicts different taxonomic levels from phylum to genus, with connecting lines representing hierarchical relationships. Each circular node represents a species; nodes colored yellow indicate no significant differences between groups, while non-yellow nodes represent characteristic microbes of the corresponding colored group (with significantly higher abundance). Color-coded sectors highlight the subordinate taxonomic ranges of characteristic microbes.

To investigate the similarities in gut microbiota among different bat species, we performed clustering analysis. At the phylum level, the differences among the three groups were not pronounced, with the majority clustering within *Firmicutes_D*, *Firmicutes_A*, and *Proteobacteria*. However, the MB group exhibited a significant clustering hotspot within *Actinobacteriota* (Fig. 2A). At the genus level to assess the similarities and differences across groups, notable clustering hotspots were identified among the three groups. The RB group predominantly clustered within *Serratia_D* (727363), *Halomonas_C* (640989), and *Citrobacter_A* (692098), alongside *Hafnia* and *Enterococcus_H* (360604), as well as *Yersinia* and *Serratia_D* (727245). The MB group primarily clustered around *Halomonas_C* (640989), *Citrobacter_A* (692098), S*erratia_D* (727363), *Alcaligenes*, *Pseudomonas_E* (650326), and *Achromobacter*. In contrast, the PB group exhibited clustering around *Hafnia*, *Enterococcus_H* (360604), *Clostridium_T*, *Paraclostridium*, *Clostridium_P*, *Lactococcus_A* (343473), and *Lactococcus_A* (346120) (Fig. 2B). At the species level, notable differences emerged in the clustering hotspots among the groups. The MB group clustered primarily around *Halomonas stevensii*, *Pseudomonas aeruginosa*, and *Alcaligenes faecalis*, whereas the clustering hotspot for the RB group was less distinct, primarily represented by *Enterococcus faecalis*. In contrast, the PB group demonstrated clustering around *Enterococcus faecalis*, *Paraclostridium benzoelyticum*, *Clostridium perfringens*, and *Clostridium sardiniense* (Fig. 2C).

Furthermore, we examined the differences in species richness among the three groups. The PB group showed a significantly higher abundance of *Firmicutes_A*, *Clostridia* (258483), *Clostridiales*, *Clostridiaceae* (222000), *Clostridium_T*, *Enterococcus_H* (360604), and *Enterococcaceae* compared to the other groups. In the MB group, *Proteobacteria*, *Gammaproteobacteria*, *Serratia_D* (727363), *Halomonas_C* (640989), *Halomonadaceae* (641030), *Pseudomonadales* (641030), *Burkholderiales* (595422), *Burkholderiaceae_A* (595422), *Achromobacter*, *Alcaligenes*, *Pseudomonadaceae*, *Pseudomonas_E* (650326), and *Pseudomonadales* (650611) were found to be more abundant than in the other groups. The RB group exhibited higher richness in *Enterobacteriaceae_A*, *Enterobacterales_A* (737866), and *Citrobacter_A* (692098) compared to the other groups (Fig. 2D). These differences and the relationships among the dominant microbial communities indicate a higher richness in the MB group, while the RB group exhibited the lowest richness (Fig. 2E).

### Alpha diversity analysis

**Fig 3:**
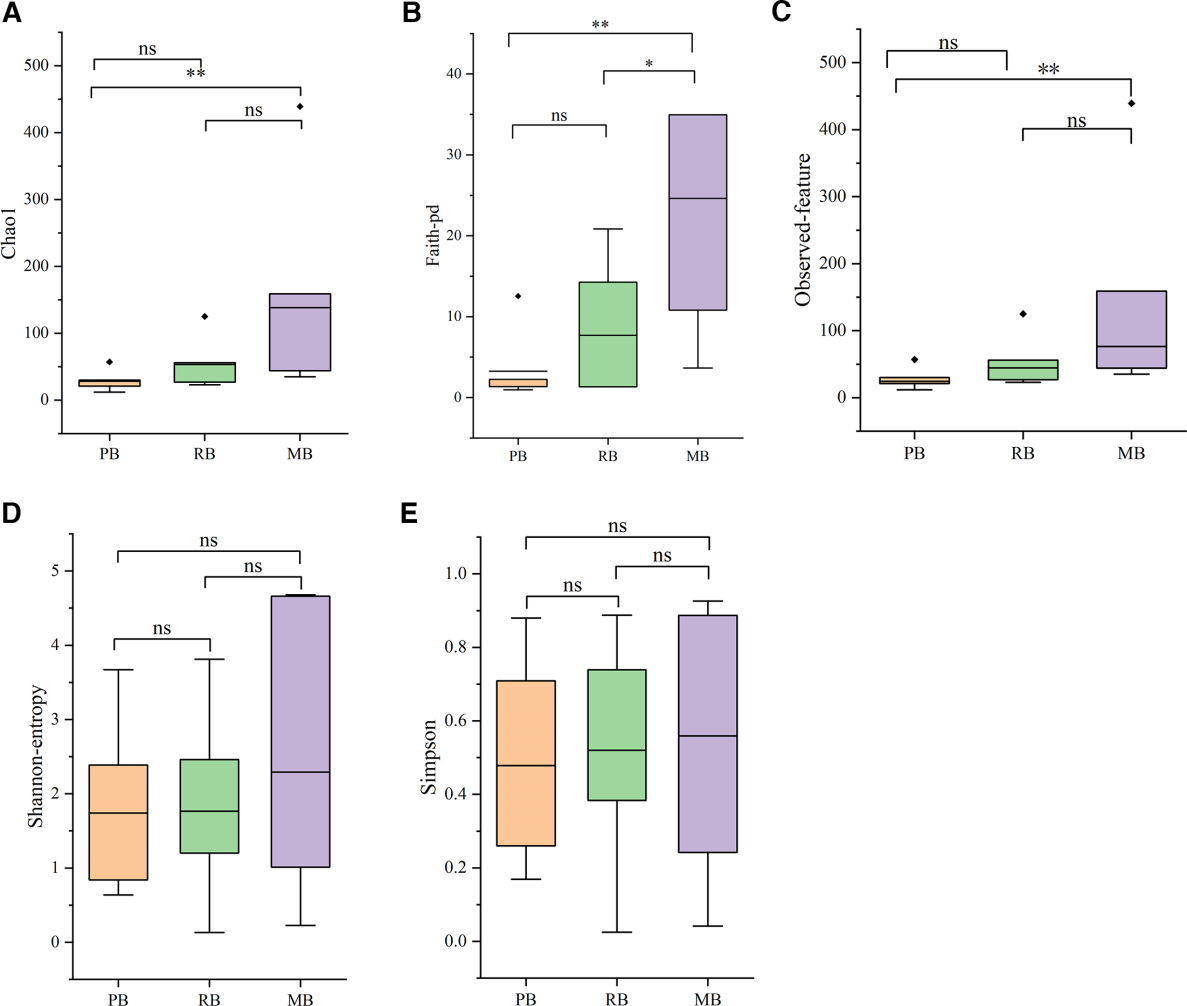
Alpha diversity analysis. (A) Results of Chao1 diversity analysis. (B) Results of Faith’s phylogenetic diversity (Faith-pd) analysis. (C) Results of observed feature diversity analysis. (D) Results of Shannon diversity analysis. (E) Results of Simpson diversity analysis. * and ** indicate *P* < 0.05 and *P* < 0.01, respectively. ◆ denotes outliers.

The results of the α-diversity analysis revealed that the species richness in the MB group was greater than that in the RB group, which in turn exceeded the richness observed in the PB group. These differences in richness were statistically significant, as indicated by the Chao1, Faith-pd, and observed feature indices (Fig. 3A through C). Although variations were also noted in the Shannon and Simpson indices, these did not reach statistical significance (Fig. 3D and E).

### Beta-diversity analysis

**Fig 4:**
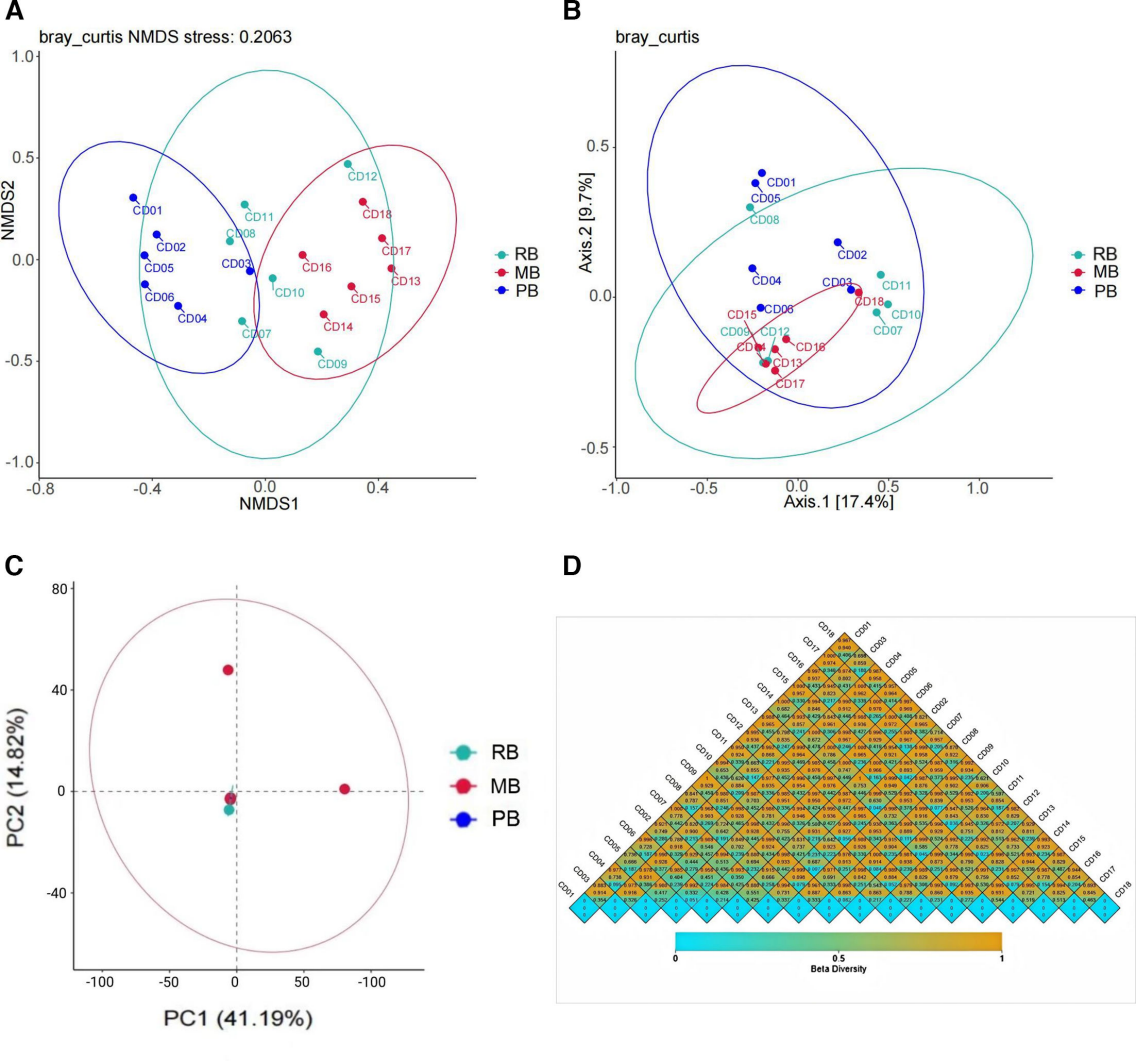
Beta diversity analysis. (A) NMDS analysis. (B) PCoA. (C) PCA. (D) Heatmap of beta diversity indices, with colors representing the dissimilarity coefficient between samples; smaller coefficients indicate greater similarity in species diversity. The numerical values in the heatmap correspond to Bray–Curtis distance, weighted UniFrac distance, and unweighted UniFrac distance, respectively.

Significant differences were observed in β-diversity among the three groups. The results from the non-metric dimensional scaling (NMDS) analysis demonstrated that the bacterial communities in the RB group were more dispersed, while the PB and MB groups exhibited higher similarity in their bacterial communities (Fig. 4A). Two-dimensional PCoA indicated that the distances between the RB and PB groups were considerably greater than those within the MB group. Moreover, the bacterial communities in the RB and PB groups appeared more dispersed, while the MB group showed greater concentration, suggesting structural differences in their communities (with a maximum variance contribution of 17.4%) (Fig. 4B). Importantly, PCA revealed that the RB and PB groups clustered together, whereas the MB group exhibited a distinct separation. This finding indicates that the bacterial compositions in the RB and PB groups are similar, while significant differences exist between MB and both RB and PB. Notably, the first principal component (PC1) was identified as the primary factor influencing bacterial diversity, contributing 41.19% to the variance (Fig. 4C).

Additionally, the analysis of Bray–Curtis, weighted UniFrac, and unweighted UniFrac distances, based on the abundance information of OTUs, was conducted to assess the differences in bacterial community structures among samples. Comparisons among the 18 samples revealed considerable variability in intestinal microbiota diversity even among individuals of the same bat species, as well as notable similarities in microbial diversity between different bat species (Fig. 4D).

### Species evolution analysis

**Fig 5:**
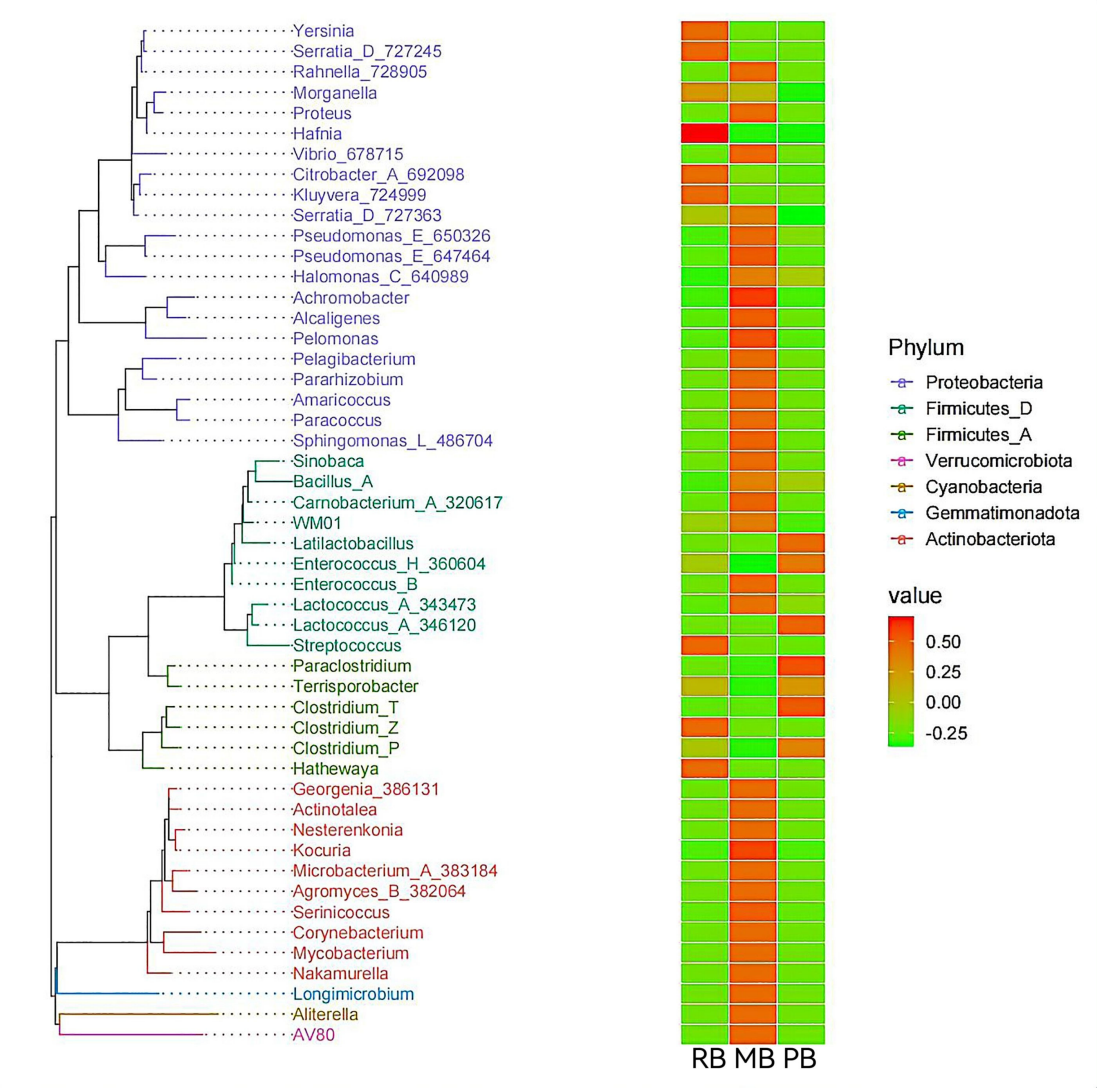
Phylogenetic tree and intergroup abundance heatmap. On the left, the phylogenetic tree with branches colored by phylum; terminal branches represent individual OTUs, annotated with their respective genera. On the right, a heatmap of standardized abundance, where higher values indicate greater relative abundance.

We selected the most abundant OTUs for each annotated genus, using these as representatives to construct a phylogenetic tree of the top 50 genera. The analysis revealed that these 50 genera belong to seven different phyla. Notably, 21 of the genera are from the phylum *Proteobacteria*, including *Yersinia, Serratia_D* (727245), *Rahnella* (728905), *Morganella*, *Proteus*, *Hafnia*, *Vibrio* (678715), *Citrobacter_A* (692098), *Kluyvera* (724999), *Serraia_D* (727363), *Pseudomonas_E* (65032), and *Pseudomonas_E* (647464). Furthermore, 10 genera are derived from the phylum *Firmicutes_D*, including *Sinobaca*, *Bacillus_A*, *Carnobacterium_A* (320617), *Latilactobacillus*, *Enterococcus_H* (360604), *Enterococcus_B*, *Lactococcus_A* (343473), *Lactococcus_A* (346120), and *Streptococcus*. Additionally, 10 genera belong to the phylum *Actinobacteriota*, comprising *Georgenia* (386131), *Actinotalea*, *Nesterenkonia*, *Kocuria*, *Microbacterium_A* (383184), *Agromyces_B* (382064), *Serinicoccus*, *Corynebacterium*, *Mycobacterium*, and *Nakamurella*. The remaining 19 genera are distributed across *Firmicutes_A*, *Verrucomicrobiota*, *Cyanobacteria*, and *Gemmatimonadota*. The MB group exhibited the highest richness hotspots, particularly within *Actinobacteriota* and *Proteobacteria*, while showing more pronounced hotspots in *Verrucomicrobiota*, *Cyanobacteria*, and *Gemmatimonadota* compared to other groups. In contrast, the RB group’s richness hotspots were primarily concentrated in *Proteobacteria* and *Firmicutes_A*. The PB group demonstrated richness hotspots predominantly in *Firmicutes_A* and *Firmicutes_D* (Fig. 5).

### Gene function prediction

**Fig 6:**
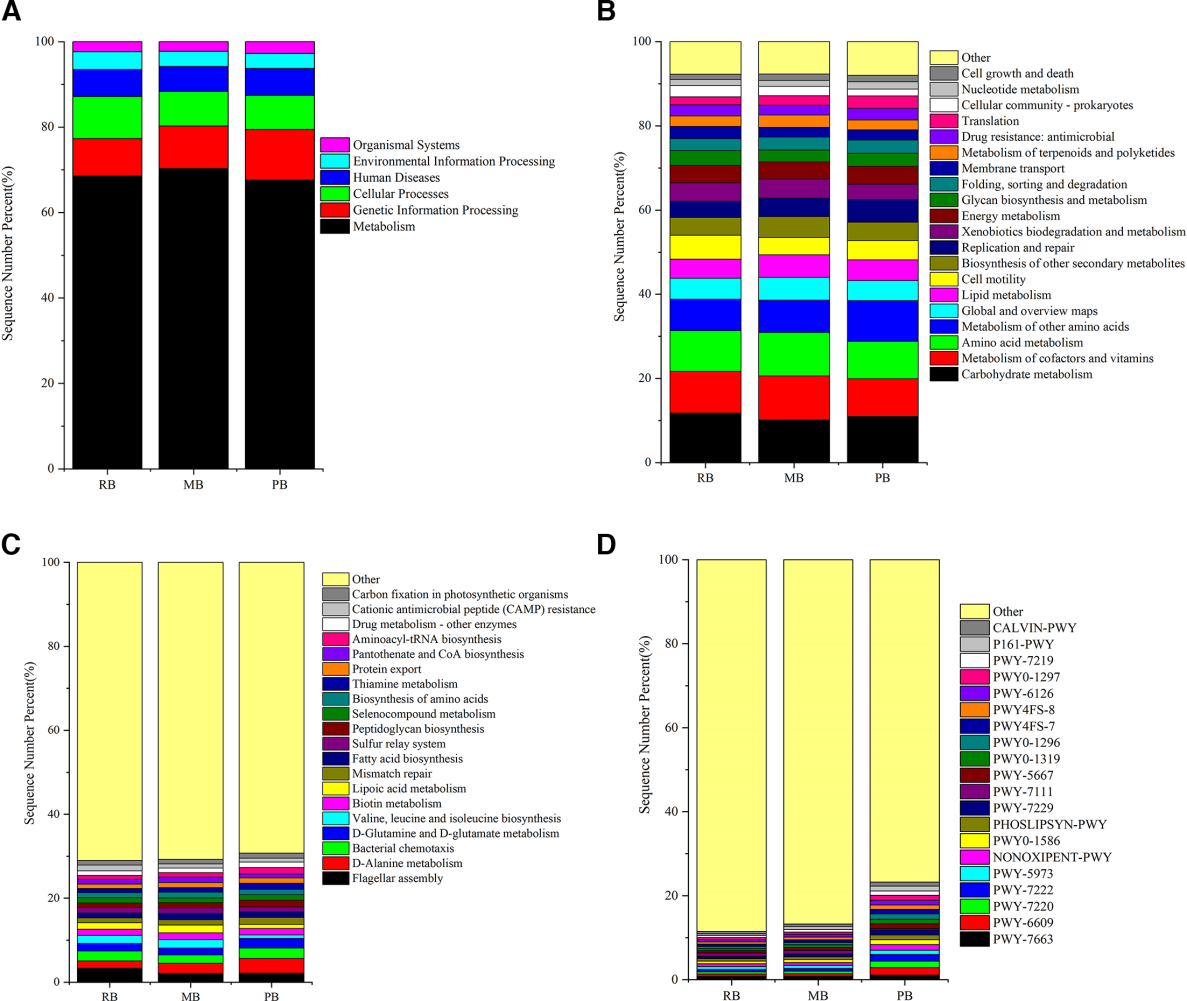
Gene function prediction. (A) Gene functions at Level 1 in the KEGG database. (B) Gene functions at Level 2 in the KEGG database (top 20). (C) Gene functions at Level 3 in the KEGG database (top 20). (D) Pathway prediction results in the MetaCyc database (top 20).

The functional predictions of gut microbiota in three bat species were conducted using the KEGG and MetaCy databases. The results revealed a total of six KEGG pathways, encompassing Metabolism, Genetic Information Processing, Cellular Processes, Human Diseases, Environmental Information Processing, and Organismal Systems. Notably, the pathway with the highest representation was Metabolism. Additionally, the Human Diseases pathway comprised 6.30% in the RB group, 5.86% in the MB group, and 6.34% in the PB group (Fig. 6A).

At the second level (L2) of the pathways, categories included Carbohydrate Metabolism, Metabolism of Cofactors and Vitamins, Amino Acid Metabolism, Metabolism of Other Amino Acids, Global and Overview Maps, Lipid Metabolism, Cell Motility, Biosynthesis of Other Secondary Metabolites, Replication and Repair, Xenobiotics Biodegradation and Metabolism, Energy Metabolism, Glycan Biosynthesis and Metabolism, Folding, Sorting and Degradation, Membrane Transport, Metabolism of Terpenoids and Polyketides, Drug Resistance: Antimicrobial, Translation, Cellular Community—Prokaryotes, Nucleotide Metabolism, and Cell Growth and Death (Fig. 6B).

The third-level pathways (L3) included D-Alanine Metabolism, Flagellar Assembly, Biosynthesis of Valine, Leucine, and Isoleucine, Bacterial Chemotaxis, Lipoic Acid Metabolism, D-Glutamine and D-Glutamate Metabolism, Biotin Metabolism, Fatty Acid Biosynthesis, Biosynthesis of Amino Acids, Sulfur Relay System, Mismatch Repair, Pantothenate and CoA Biosynthesis, Peptidoglycan Biosynthesis, Protein Export, Selenocompound Metabolism, Thiamine Metabolism, Aminoacyl-tRNA Biosynthesis, Carbon Fixation in Photosynthetic Organisms, Drug Metabolism—Other Enzymes, and Cationic Antimicrobial Peptide (CAMP) Resistance (Fig. 6C).

The pathways predicted by the MetaCy database included PWY-6609, PWY-7220, PWY-7222, PWY0-1296, NONOXIPENT-PWY, PWY0-1297, PWY0-1586, P161-PWY, PWY-7229, PHOSLIPSYN-PWY, PWY-7663, PWY-6126, PWY-5667, PWY0-1319, PWY4FS-7, PWY4FS-8, PWY-5973, PWY-7219, CALVIN-PWY, and PWY-7111. Among these, PWY-6609, PWY-7220, PWY-7222, PWY0-1296, NONOXIPENT-PWY, and PWY0-1297 showed a higher representation in the PB group (Fig. 6D).

**Fig 7:**
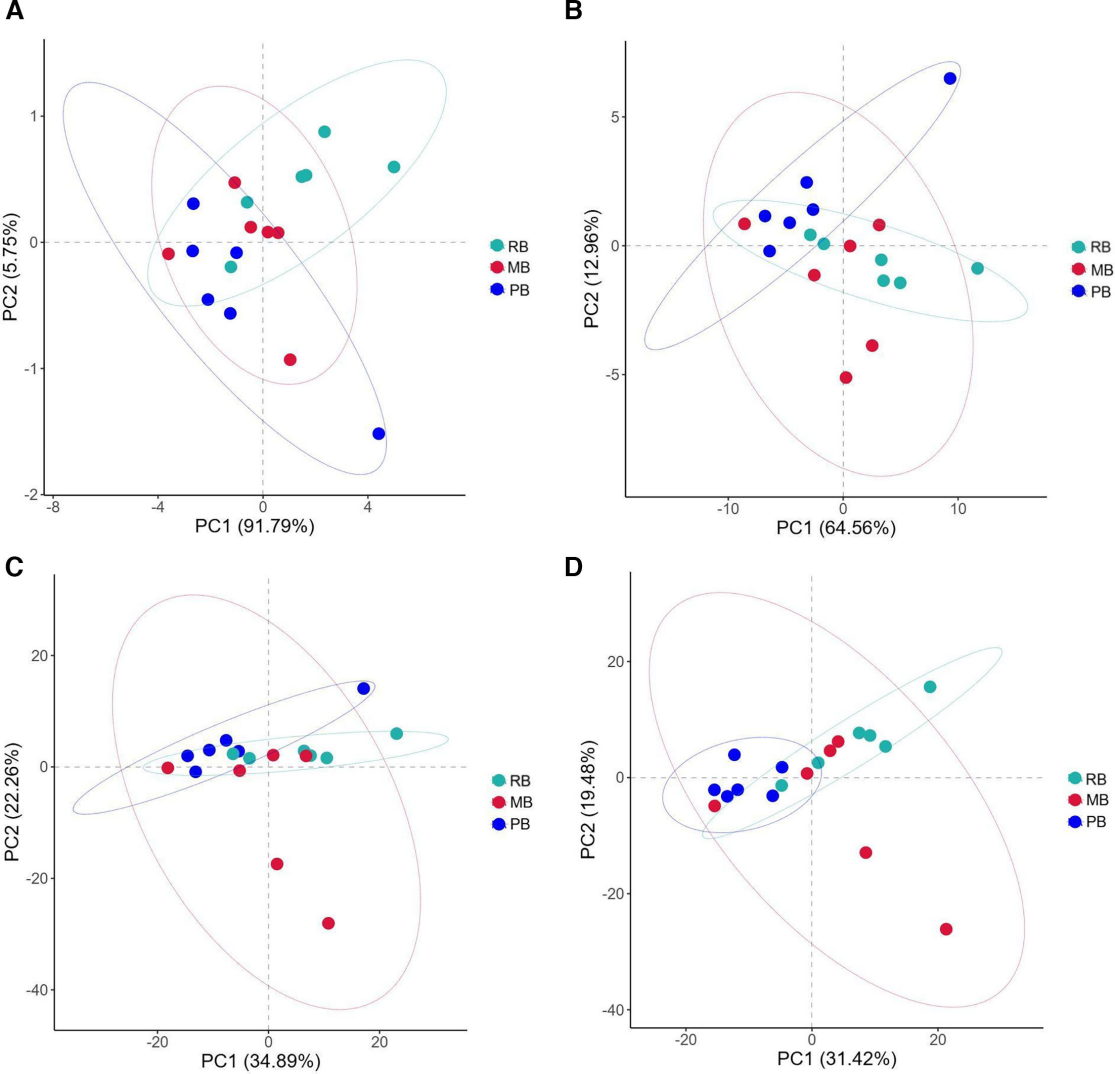
Predictive functional PCA. (A) PCA of KEGG pathways at the L1 level. (B) PCA of KEGG pathways at the L2 level. (C) PCA of KEGG pathways at the L3 level. (D) PCA of MetaCyc pathways.

PCA was performed on the predicted gene functions, revealing significant differences among the three groups. At the L1 level of KEGG pathways, these differences were not pronounced, with PC1 accounting for 91.79% of the variation among samples (Fig. 7A). In contrast, at the L2 and L3 levels of KEGG pathways, the RB and PB groups exhibited similarity and concentrated gene functions, while the MB group demonstrated a broader range of gene functions (Fig. 7B and C). These results were also evident in the MetaCyc pathways, highlighting the distinct gene functional characteristics of the MB group (Fig. 7D).

### Culturomics results

**Fig 8:**
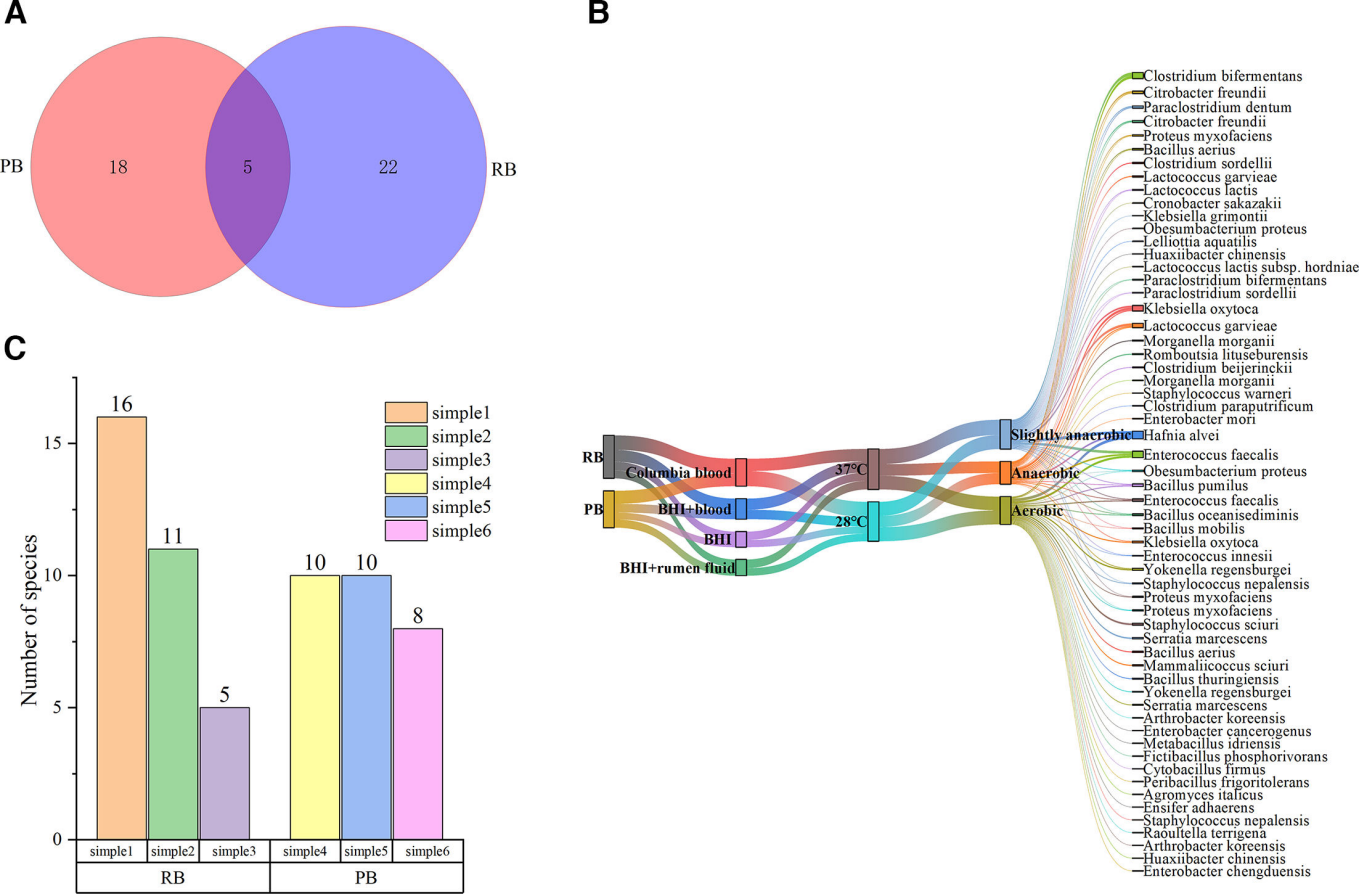
Results of culturomics. (A) Venn diagram of cultured bacterial species. (B) Bacterial species grown under different culturing conditions. (C) Number of species isolated from each sample.

The cultivation results from the PB and RB groups revealed that 221 suspected colonies were captured from six intestinal samples under various media and conditions. Among the 221 colonies identified using MALDI-TOF MS, 124 exhibited scores greater than 9 (Table S2). Furthermore, sequence alignments of the 16S rRNA gene from 97 strains indicated that 96 had similarity values exceeding 99% in the NCBI database (Table S3). Notably, one strain showed only 98.18% similarity to *Clostridium paraputrificum* strain JCM 1293 (Table S4), suggesting it may represent a putative new species. For new bacterial species identification, average nucleotide identity (ANI) and digital DNA–DNA hybridization (dDDH) are regarded as the gold standards. Thresholds for ANI and dDDH were set at 95% and 70%, respectively, with values below these thresholds indicating that the strains belong to a new species (28–30). To this end, we performed whole-genome sequencing of the putative new species, and the ANI and dDDH analyses confirmed that it represents a new species of *Clostridium* (ANI below 95%; dDDH below 70%). The detailed validation process will be reported in a forthcoming publication. In total, by integrating the results from MALDI-TOF MS and 16S rRNA gene sequencing, we successfully cultured 45 bacterial species. Specifically, the PB group yielded 23 distinct species, while the RB group produced 27 species, with only five species being common to both groups (Fig. 8A). The most abundant cultured bacterial species included *Enterococcus faecalis*, *Hafnia alvei*, *Klebsiella oxytoca*, *Clostridium bifermentans*, *Lactococcus garvieae*, *Citrobacter freundii*, *Proteus myxofaciens*, and *Yokenella regensburgei*. Additionally, several bacterial species that have been recently identified and may pose potential health risks to humans were noted, such as *Enterobacter chengduensis*, *Huaxiibacter chinensis*, and *Morganella morganii* (Fig. 8B). Among the culture media, the Columbia blood agar demonstrated a significant predominance, accounting for 34.39% of the isolates, followed by BHI supplemented with blood at 25.79%. The growth rates at 28°C and 37°C were similar, comprising 49.32% and 50.68% of the total, respectively. Slightly anaerobic conditions yielded the highest success rate at 36.65%, followed closely by aerobic conditions at 35.29%, whereas anaerobic conditions resulted in the lowest recovery rate at 28.05% (Fig. 8B). Notably, the RB group exhibited the greatest variability in bacterial counts, with sample 1 yielding 16 species and sample 3 producing only five species. In contrast, the PB group showed minimal variation in the number of bacterial species cultivated across samples (Fig. 8C).

## DISCUSSION

This study offers a comprehensive examination of the gut microbiota of hibernating bats in Guizhou Province, China, utilizing high-throughput sequencing and culture-based methods. Given the ecological significance of bats as vectors of various pathogens and their role in maintaining ecosystem balance, understanding their gut microbiota can provide valuable insights into host health, metabolic functions, and the potential for zoonotic disease transmission.

Our findings demonstrate distinct differences in the gut microbiota composition among the three bat species examined: the PB, MB, and RB. At the phylum level, *Proteobacteria* emerged as the predominant group across all three bat cohorts, albeit with notable differences in the relative abundance. The RB group exhibited the highest percentage of *Proteobacteria* (80.99%), followed by MB (60.37%) and PB (14.76%). This finding is consistent with those of previous studies demonstrating the dominance of *Proteobacteria* in various mammalian gut microbiomes, which has been associated with their roles in nutrient metabolism and immune modulation (31, 32). In contrast, *Firmicutes*, particularly *Firmicutes_A* and *Firmicutes_D*, were notably more abundant in the PB group, suggesting a unique adaptation to their diet or gut physiology (33, 34). At the genus level, our analysis revealed significant microbial diversity. The RB group’s high abundance of *Hafnia* (40.44%) and *Yersinia* (12.91%) may reflect their dietary preferences or environmental exposures, given that *Hafnia* is often found in decaying matter and can thrive in varied habitats, and *Yersinia* poses a serious threat to human health. These include *Yersinia pestis*, *Yersinia enterocolitica*, and *Yersinia pseudotuberculosis* (35–37). The presence of *Enterococcus* in both RB and PB groups further underscores the potential importance of this genus in gut health and disease prevention (38, 39). The difference is even greater at the species level; the clustering analysis based on the top 20 genera underscores distinct microbiota profiles among the three groups. The identification of unique clustering patterns in RB, MB, and PB suggests that specific environmental or dietary factors significantly influence the microbial community structure. Moreover, the high proportion of unclassified OTUs across all groups, particularly in the RB (83.81%) and MB (69.35%) cohorts, suggests that a significant portion of the gut microbiota remains uncharacterized, warranting further investigation into these unidentified taxa and their potential roles. This underscores the necessity for more comprehensive metagenomic and culturomics studies to elucidate the functional capacities of these microbial communities. The analysis of OTU richness revealed a noteworthy disparity among the groups, with MB hosting the most OTUs (729), followed by RB (226) and PB (126). This suggests a more diverse and potentially resilient microbial ecosystem in MB, which could be a reflection of varied dietary inputs or environmental interactions (3–6). The shared OTUs among all three groups (22) indicate a core microbiota, possibly essential for basic gut functions, while the unique OTUs may contribute to group-specific adaptations.

Obviously, the intestinal microbial diversity of MB is higher than that of RB, and the least is PB. This suggests that dietary preferences and environmental factors could significantly shape the microbiota composition within these species (4–6). It also suggests that different species of bats may pose different threats to humans because of differences in the bacteria they carry in their guts. It is also worth noting that, compared with non-hibernating bats, whether there are differences in the number of intestinal microbes and species diversity in hibernating bats deserves further study (3).

Our analysis of α-diversity indicated that the MB group harbors a higher species richness compared to both the RB and PB groups, with statistically significant differences observed in the Chao1, Faith-pd, and observed feature indices. These indices are commonly used to estimate species richness and phylogenetic diversity, and the observed trends suggest that MB bats support a more diverse microbial community. The significant differences in richness between groups may reflect variations in diet, habitat, or other ecological factors that influence gut microbiota composition. In particular, microbats, which tend to have a more insect-based diet, might host a distinct set of microbes compared to other bat species with different feeding strategies (e.g., fruit or nectar). This hypothesis is supported by studies in other mammals, where dietary habits have been shown to shape gut microbial communities (5, 6). Interestingly, although Shannon and Simpson indices revealed trends toward differences in community evenness and diversity, no statistically significant differences were found between groups. This result suggests that while the species richness differs significantly between groups, the evenness of species distribution within each group does not show a comparable level of variation. Our β-diversity analysis further highlighted the distinct microbial community structures across the three bat groups. Even though all three bat species come from the same area, NMDS and PCoA clearly indicated that the RB group exhibited a more dispersed bacterial community, while the MB and PB groups showed a higher degree of microbial similarity. This observation suggests that the microbiomes of MB and PB bats are more alike, potentially due to shared ecological niches or overlapping environmental exposures, which are less apparent in the RB group (40–42). Furthermore, the PCA revealed that RB and PB bats clustered more tightly together, while MB bats were more clearly separated, suggesting that the microbial communities in the MB group are distinct from those in the other groups. This separation could be attributed to specific ecological factors or host traits, such as feeding behavior, roosting environment, or microbiota-host co-evolution, which have been shown to play a significant role in shaping the microbial diversity in wildlife (43–45). Notably, PC1 accounted for the largest proportion of variance in community structure (41.19%), indicating that this principal component is the most significant factor contributing to the microbial diversity observed among these bat species. The Bray–Curtis, Weighted Unifrac, and Unweighted Unifrac distance metrics, which evaluate the compositional differences between samples, confirmed the distinct microbial profiles between the bat groups. Interestingly, we observed that not only did the interspecies microbial differences vary, but within-species variability also played a significant role. This finding supports the hypothesis that gut microbiota composition can exhibit high levels of intra-species variability, even among individuals of the same species (46–48). These results align with those of previous studies in other wild mammals, which have also found considerable gut microbiota variation within species, potentially influenced by factors such as age, sex, health status, and environmental exposure (49–51). Importantly, our study also revealed that different bat species could exhibit similar microbiota profiles, despite being distinct species. This suggests that ecological factors such as diet or habitat, rather than genetic differences alone, may drive microbial similarities across species.

The analysis of the dominant OTUs within our study revealed a rich diversity among the bacterial communities identified across various groups. The 50 predominant genera, representing seven distinct phyla, underscore the complexity and ecological significance of these microbial populations. Notably, the prevalence of *Proteobacteria* with 21 genera, including *Yersinia*, *Serratia*, and *Pseudomonas*, may pose a threat to human health. Our results demonstrate that the MB group harbors the highest richness hotspots, particularly in *Actinobacteriota* and *Proteobacteria*. This finding may reflect specific environmental conditions favoring these taxa, such as nutrient availability and competitive interactions (52, 53). The pronounced hotspots in *Verrucomicrobiota*, *Cyanobacteria*, and *Gemmatimonadota* suggest unique ecological niches that warrant further investigation as these phyla are less frequently highlighted in microbial diversity studies. The distinctive profiles of the RB and PB groups, with hotspots concentrated in *Proteobacteria* and *Firmicutes*, respectively, indicate differing ecological strategies and adaptations to their environments.

The gut microbiome is pivotal in maintaining host health and influencing various physiological processes, including metabolism and immune response. Our findings, derived from KEGG and MetaCy databases, reveal significant insights into the functional capabilities of gut microbial communities in three bat species. The functional prediction of gut microbiota based on KEGG pathway analysis highlighted six major categories, with Metabolism being the most prevalent. This aligns with previous research suggesting that metabolic pathways are fundamental to the role of gut microbiota in nutrient absorption and digestion (54–56). The high percentage of Human Diseases pathways (6.30% in RB, 5.86% in MB, and 6.34% in PB) underscores the potential impact of the gut microbiome on host health, particularly concerning its association with metabolic disorders, autoimmune diseases, and other health issues (57, 58). The second-level pathways revealed extensive metabolic diversity, including Carbohydrate metabolism, Amino acid metabolism, and Lipid metabolism. The presence of diverse carbohydrate metabolism pathways may suggest that these bats have adapted to exploit a variety of dietary sources, potentially aiding in their ecological flexibility (59–61). At the third-level pathway analysis, the identification of pathways like D-Alanine metabolism and Valine, leucine, and isoleucine biosynthesis indicates that these bats possess specialized microbial functions that might contribute to protein synthesis and neurotransmitter production, further influencing metabolic health (62, 63). Our comprehensive functional analysis of the gut microbiota across three bat species provides essential insights into the metabolic capabilities and potential health implications associated with these microbial communities. The significant presence of pathways related to metabolism and human diseases emphasizes the role of gut microbiota in ecological and evolutionary contexts. Future studies should focus on longitudinal assessments of gut microbiome functionality to unravel the dynamic interactions between diet, gut microbiota, and host health. These findings not only contribute to our understanding of bat ecology but also offer a framework for exploring gut microbiome functionality in other wildlife and its implications for conservation biology.

Culturomics studies have yielded important results, and the differential abundance of bacterial species between the PB and RB groups suggests species-specific microbiota composition. The isolation of 23 and 27 bacterial species from PB and RB, respectively, with only five species shared between the two groups, indicates a distinct microbial niche that may be influenced by factors such as diet, habitat, and behavior (40–42). The results of the culture-based analysis also revealed the presence of recently reported bacterial species with potential pathogenicity to humans, specifically *Huaxiibacter chinensis* and *Enterobacter chengduensis* (64, 65). Based on prior research experience, a 16S rRNA gene sequence similarity below 98.65% is often indicative of a potential new species. We appear to have identified a novel bacterial species that is closely related to *Clostridium paraputrificum*. We performed whole-genome sequencing of the strain, and the ANI and dDDH analyses confirmed that it represents a new species of *Clostridium* (ANI below 95%, dDDH below 70%). The detailed validation process will be reported in a forthcoming publication (66). This study marks the first identification of these species in bats, providing valuable insights for tracing the transmission pathways and epidemiological research related to these bacteria’s potential infection routes in humans. The higher diversity observed in the RB group, particularly in sample 1, which yielded 16 species compared to only five in sample 3, points to potential environmental or physiological factors affecting bacterial colonization and growth. In addition, we found that the cultivation results further revealed that the Columbia blood agar plate was the most successful medium. The marginal differences in bacterial growth at 28°C and 37°C suggest that these bat-associated bacteria are adapted to a range of thermal conditions, reflecting their ecological versatility. Furthermore, the success of slightly anaerobic conditions (36.65%) over strictly aerobic (35.29%) and anaerobic (28.05%) environments may imply a predominance of facultative anaerobes within the bat gut microbiota. Culturomics is essential for studying the effects of microorganisms on human health and is complementary to high-throughput sequencing (67, 68). Although different temperatures, different oxygen levels, and different nutritional conditions can produce different types of intestinal bacteria, it is necessary to set as many culture conditions as possible when carrying out large-scale bacterial culture because there are always a few special bacteria that prefer certain special conditions.

Future investigations will incorporate expanded sampling to enhance statistical power. Subsequent studies will explicitly address environmental influences on bat microbiomes, particularly the unique biogeographical and climatic features characterizing Guizhou’s karst landscapes.

### Conclusion

Our research reveals significant differences in the composition and diversity of the gut microbiota among three bat groups (PB, MB, and RB) from Guizhou. While *Proteobacteria* predominates in all groups, its abundance varies. Notably, the high richness of OTUs in the MB group suggests a more diverse microbial ecosystem, underscoring the complex interactions between species diversity, diet, gut microbiota, and overall ecological dynamics in bats. Furthermore, the substantial presence of unknown bacterial species in their intestines highlights the critical importance of cultivation-based approaches. The presence of specific taxa may have potential health implications for both bats and humans. These findings emphasize the need for further investigations into the functional roles of these microbiota and their contributions to host health. Future research should focus on longitudinal studies to elucidate these intricate interactions.

## Data Availability

All the raw data are analyzed, and the script has been uploaded to Figshare: https://doi.org/10.6084/m9.figshare.27274665.

## SUPPLEMENTAL MATERIAL

TABLE S1 msystems.01463-24-s0001.xlsx Basic information and environmental parameters of bat samples.

TABLE S2 msystems.01463-24-s0002.xlsx Results of MALDI-TOF MS identification.

TABLE S3 msystems.01463-24-s0003.xlsx Results of 16S rRNA gene sequence identification.

TABLE S4 msystems.01463-24-s0004.xlsx 16S rRNA gene sequence of suspected new species.

ASM does not own the copyrights to Supplemental Material that may be linked to, or accessed through, an article. The authors have granted ASM a non-exclusive, world-wide license to publish the Supplemental Material files. Please contact the corresponding author directly for reuse.

## References

[1] Castelo-Branco D, Nobre JA, Souza PRH, Diógenes EM, Guedes GMM, Mesquita FP, Souza PFN, Rocha MFG, Sidrim JJC, Cordeiro RA, Montenegro RC. 2023. Role of Brazilian bats in the epidemiological cycle of potentially zoonotic pathogens. Microb Pathog 177:106032. doi:10.1016/j.micpath.2023.106032 36804526

[2] Federici L, Masulli M, De Laurenzi V, Allocati N. 2022. An overview of bats microbiota and its implication in transmissible diseases. Front Microbiol 13:1012189. doi:10.3389/fmicb.2022.1012189 36338090 PMC9631491

[3] Xiao G, Liu S, Xiao Y, Zhu Y, Zhao H, Li A, Li Z, Feng J. 2019. Seasonal changes in gut microbiota diversity and composition in the greater horseshoe bat. Front Microbiol 10:2247. doi:10.3389/fmicb.2019.02247 31632369 PMC6779692

[4] Liu S, Xiao Y, Wang X, Guo D, Wang Y, Wang Y. 2023. Effects of microhabitat temperature variations on the gut microbiotas of free-living hibernating animals. Microbiol Spectr 11:e00433-23. doi:10.1128/spectrum.00433-23 37378560 PMC10434193

[5] Dai W, Leng H, Li J, Li A, Li Z, Zhu Y, Li X, Jin L, Sun K, Feng J. 2024. The role of host traits and geography in shaping the gut microbiome of insectivorous bats. mSphere 9:e00087-24. doi:10.1128/msphere.00087-24 38509042 PMC11036801

[6] Li J, Li L, Jiang H, Yuan L, Zhang L, Ma J-E, Zhang X, Cheng M, Chen J. 2018. Fecal bacteriome and mycobiome in bats with diverse diets in south China. Curr Microbiol 75:1352–1361. doi:10.1007/s00284-018-1530-0 29922970

[7] Zheng W, Zhao S, Yin Y, Zhang H, Needham DM, Evans ED, Dai CL, Lu PJ, Alm EJ, Weitz DA. 2022. High-throughput, single-microbe genomics with strain resolution, applied to a human gut microbiome. Science 376. doi:10.1126/science.abm1483 35653470

[8] Swanson KS, de Vos WM, Martens EC, Gilbert JA, Menon RS, Soto-Vaca A, Hautvast J, Meyer PD, Borewicz K, Vaughan EE, Slavin JL. 2020. Effect of fructans, prebiotics and fibres on the human gut microbiome assessed by 16S rRNA-based approaches: a review. Benef Microbes 11:101–129. doi:10.3920/BM2019.0082 32073295

[9] Jin J, Yamamoto R, Shiroguchi K. 2024. High-throughput identification and quantification of bacterial cells in the microbiota based on 16S rRNA sequencing with single-base accuracy using BarBIQ. Nat Protoc 19:207–239. doi:10.1038/s41596-023-00906-8 38012397

[10] O’Toole PW, Flemer B. 2017. From culture to high-throughput sequencing and beyond: a layperson's guide to the "Omics" and diagnostic potential of the microbiome. Gastroenterol Clin North Am 46:9–17. doi:10.1016/j.gtc.2016.09.003 28164855

[11] Gutleben J, Chaib De Mares M, van Elsas JD, Smidt H, Overmann J, Sipkema D. 2018. The multi-omics promise in context: from sequence to microbial isolate. Crit Rev Microbiol 44:212–229. doi:10.1080/1040841X.2017.1332003 28562180

[12] Salama ES, Govindwar SP, Khandare RV, Roh HS, Jeon BH, Li X. 2019. Can omics approaches improve microalgal biofuels under abiotic stress? Trends Plant Sci 24:611–624. doi:10.1016/j.tplants.2019.04.001 31085124

[13] Cui J, Li F, Shi Z-L. 2019. Origin and evolution of pathogenic coronaviruses. Nat Rev Microbiol 17:181–192. doi:10.1038/s41579-018-0118-9 30531947 PMC7097006

[14] Fung TS, Liu DX. 2019. Human Coronavirus: host-pathogen interaction. Annu Rev Microbiol 73:529–557. doi:10.1146/annurev-micro-020518-115759 31226023

[15] Callahan BJ, McMurdie PJ, Rosen MJ, Han AW, Johnson AJA, Holmes SP. 2016. DADA2: high-resolution sample inference from Illumina amplicon data. Nat Methods 13:581–583. doi:10.1038/nmeth.3869 27214047 PMC4927377

[16] Caporaso JG, Kuczynski J, Stombaugh J, Bittinger K, Bushman FD, Costello EK, Fierer N, Peña AG, Goodrich JK, Gordon JI, et al.. 2010. QIIME allows analysis of high-throughput community sequencing data. Nat Methods 7:335–336. doi:10.1038/nmeth.f.303 20383131 PMC3156573

[17] Lagier J-C, Armougom F, Million M, Hugon P, Pagnier I, Robert C, Bittar F, Fournous G, Gimenez G, Maraninchi M, Trape J-F, Koonin EV, La Scola B, Raoult D. 2012. Microbial culturomics: paradigm shift in the human gut microbiome study. Clin Microbiol Infect 18:1185–1193. doi:10.1111/1469-0691.12023 23033984

[18] Lagier J-C, Hugon P, Khelaifia S, Fournier P-E, La Scola B, Raoult D. 2015. The rebirth of culture in microbiology through the example of culturomics to study human gut microbiota. Clin Microbiol Rev 28:237–264. doi:10.1128/CMR.00014-14 25567229 PMC4284300

[19] Tsuchida S, Umemura H, Nakayama T. 2020. Current status of matrix-assisted laser desorption/Ionization-time-of-flight mass spectrometry (MALDI-TOF MS) in clinical diagnostic microbiology. Molecules 25:4775. doi:10.3390/molecules25204775 33080897 PMC7587594

[20] Ashfaq MY, Da’na DA, Al-Ghouti MA. 2022. Application of MALDI-TOF MS for identification of environmental bacteria: a review. J Environ Manage 305:114359. doi:10.1016/j.jenvman.2021.114359 34959061

[21] Jeong JH, Kweon OJ, Kim HR, Kim T-H, Ha S-M, Lee M-K. 2021. A novel species of the genus Arsenicicoccus isolated from human blood using whole-genome sequencing. Ann Lab Med 41:323–327. doi:10.3343/alm.2021.41.3.323 33303718 PMC7748104

[22] Khanal M, Timilsina S, Bhatta BP, Bophela K, Coutinho T, Cochran K, Malla S. 2022. Pseudomonas uvaldensis sp. nov., a bacterial pathogen causing onion bulb rot. Int J Syst Evol Microbiol 72. doi:10.1099/ijsem.0.005311 35442877

[23] McDonald D, Jiang Y, Balaban M, Cantrell K, Zhu Q, Gonzalez A, Morton JT, Nicolaou G, Parks DH, Karst SM, Albertsen M, Hugenholtz P, DeSantis T, Song SJ, Bartko A, Havulinna AS, Jousilahti P, Cheng S, Inouye M, Niiranen T, Jain M, Salomaa V, Lahti L, Mirarab S, Knight R. 2024. Greengenes2 unifies microbial data in a single reference tree. Nat Biotechnol 42:715–718. doi:10.1038/s41587-023-01845-1 37500913 PMC10818020

[24] Segata N, Izard J, Waldron L, Gevers D, Miropolsky L, Garrett WS, Huttenhower C. 2011. Metagenomic biomarker discovery and explanation. Genome Biol 12:R60. doi:10.1186/gb-2011-12-6-r60 21702898 PMC3218848

[25] Yu G. 2020. Using ggtree to visualize data on tree-like structures. CP in Bioinformatics 69:e96. doi:10.1002/cpbi.96 32162851

[26] Douglas GM, Maffei VJ, Zaneveld JR, Yurgel SN, Brown JR, Taylor CM, Huttenhower C, Langille MGI. 2020. PICRUSt2 for prediction of metagenome functions. Nat Biotechnol 38:685–688. doi:10.1038/s41587-020-0548-6 32483366 PMC7365738

[27] Gao Y, Zhang G, Jiang S, Liu Y-X. 2024. Wekemo bioincloud: a user-friendly platform for meta-omics data analyses. Imeta 3:e175. doi:10.1002/imt2.175 38868508 PMC10989175

[28] Diop A, El Karkouri K, Raoult D, Fournier PE. 2020. Genome sequence-based criteria for demarcation and definition of species in the genus Rickettsia. Int J Syst Evol Microbiol 70:1738–1750. doi:10.1099/ijsem.0.003963 31935173

[29] Kook J-K, Park S-N, Lim YK, Cho E, Jo E, Roh H, Shin Y, Paek J, Kim H-S, Kim H, Shin JH, Chang Y-H. 2017. Genome-based reclassification of Fusobacterium nucleatum subspecies at the species level. Curr Microbiol 74:1137–1147. doi:10.1007/s00284-017-1296-9 28687946

[30] Alotaibi F, Lee SJ, Lahrach Z, St-Arnaud M, Hijri M. 2023. Draft genome of Nocardia canadensis sp. nov. isolated from petroleum-hydrocarbon-contaminated soil. Microorganisms 11:2972. doi:10.3390/microorganisms11122972 38138115 PMC10745995

[31] Das B, Ghosh TS, Kedia S, Rampal R, Saxena S, Bag S, Mitra R, Dayal M, Mehta O, Surendranath A, Travis SPL, Tripathi P, Nair GB, Ahuja V. 2018. Analysis of the gut microbiome of rural and urban healthy indians living in sea level and high altitude areas. Sci Rep 8:10104. doi:10.1038/s41598-018-28550-3 29973712 PMC6031670

[32] Gureev AP, Shaforostova EA, Vitkalova IYu, Sadovnikova IS, Kalinina YI, Cherednichenko VR, Reznikova KA, Valuyskikh VV, Popov VN. 2020. Long-term mildronate treatment increased Proteobacteria level in gut microbiome, and caused behavioral deviations and transcriptome change in liver, heart and brain of healthy mice. Toxicol Appl Pharmacol 398:115031. doi:10.1016/j.taap.2020.115031 32389661

[33] Sun Y, Zhang S, Nie Q, He H, Tan H, Geng F, Ji H, Hu J, Nie S. 2023. Gut firmicutes: relationship with dietary fiber and role in host homeostasis. Crit Rev Food Sci Nutr 63:12073–12088. doi:10.1080/10408398.2022.2098249 35822206

[34] Bensch HM, Tolf C, Waldenström J, Lundin D, Zöttl M. 2023. Bacteroidetes to firmicutes: captivity changes the gut microbiota composition and diversity in a social subterranean rodent. Anim Microbiome 5:9. doi:10.1186/s42523-023-00231-1 36765400 PMC9912604

[35] Barbieri R, Signoli M, Chevé D, Costedoat C, Tzortzis S, Aboudharam G, Raoult D, Drancourt M. 2020. Yersinia pestis: the natural history of plague. Clin Microbiol Rev 34:e00044-19. doi:10.1128/CMR.00044-19 33298527 PMC7920731

[36] Seabaugh JA, Anderson DM. 2024. Pathogenicity and virulence of Yersinia. Virulence 15:2316439. doi:10.1080/21505594.2024.2316439 38389313 PMC10896167

[37] Mecsas J. 2019. Unraveling neutrophil- Yersinia interactions during tissue infection. F1000Res 32:8. doi:10.1128/CMR.00058-18 PMC6625543 31327994

[38] García-Solache M, Rice LB. 2019. The Enterococcus: a model of adaptability to its environment. Clin Microbiol Rev 32:e00058-18. doi:10.1128/CMR.00058-18 30700430 PMC6431128

[39] Yuen GJ, Ausubel FM. 2014. Enterococcus infection biology: lessons from invertebrate host models. J Microbiol 52:200–210. doi:10.1007/s12275-014-4011-6 24585051 PMC4556283

[40] Smith AB, Godsoe W, Rodríguez-Sánchez F, Wang H-H, Warren D. 2019. Niche estimation above and below the species level. Trends Ecol Evol 34:260–273. doi:10.1016/j.tree.2018.10.012 30497791

[41] Picciotti U, Araujo Dalbon V, Ciancio A, Colagiero M, Cozzi G, De Bellis L, Finetti-Sialer MM, Greco D, Ippolito A, Lahbib N, Logrieco AF, López-Llorca LV, Lopez-Moya F, Luvisi A, Mincuzzi A, Molina-Acevedo JP, Pazzani C, Scortichini M, Scrascia M, Valenzano D, Garganese F, Porcelli F. 2023. “Ectomosphere”: insects and microorganism interactions. Microorganisms 11:440. doi:10.3390/microorganisms11020440 36838405 PMC9967823

[42] Fagre AC, Kading RC. 2019. Can bats serve as reservoirs for arboviruses? Viruses 11:215. doi:10.3390/v11030215 30832426 PMC6466281

[43] Ayres JS. 2016. Cooperative microbial tolerance behaviors in host-microbiota mutualism. Cell 165:1323–1331. doi:10.1016/j.cell.2016.05.049 27259146 PMC4903080

[44] Forde B, Yao L, Shaha R, Murphy S, Lunjani N, O’Mahony L. 2022. Immunomodulation by foods and microbes: unravelling the molecular tango. Allergy 77:3513–3526. doi:10.1111/all.15455 35892227 PMC10087875

[45] Vlasova AN, Takanashi S, Miyazaki A, Rajashekara G, Saif LJ. 2019. How the gut microbiome regulates host immune responses to viral vaccines. Curr Opin Virol 37:16–25. doi:10.1016/j.coviro.2019.05.001 31163292 PMC6863389

[46] Zhu A, Sunagawa S, Mende DR, Bork P. 2015. Inter-individual differences in the gene content of human gut bacterial species. Genome Biol 16:82. doi:10.1186/s13059-015-0646-9 25896518 PMC4428241

[47] Greenblum S, Carr R, Borenstein E. 2015. Extensive strain-level copy-number variation across human gut microbiome species. Cell 160:583–594. doi:10.1016/j.cell.2014.12.038 25640238 PMC4507803

[48] Wu Y, Zheng Y, Wang S, Chen Y, Tao J, Chen Y, Chen G, Zhao H, Wang K, Dong K, Hu F, Feng Y, Zheng H. 2022. Genetic divergence and functional convergence of gut bacteria between the Eastern honey bee Apis cerana and the Western honey bee Apis mellifera. J Adv Res 37:19–31. doi:10.1016/j.jare.2021.08.002 35499050 PMC9039653

[49] Brown K, Thomson CA, Wacker S, Drikic M, Groves R, Fan V, Lewis IA, McCoy KD. 2023. Microbiota alters the metabolome in an age- and sex- dependent manner in mice. Nat Commun 14:1348. doi:10.1038/s41467-023-37055-1 36906623 PMC10008592

[50] Korteniemi J, Karlsson L, Aatsinki A. 2023. Systematic review: autism spectrum disorder and the gut microbiota. Acta Psychiatr Scand 148:242–254. doi:10.1111/acps.13587 37395517

[51] Sivamaruthi BS, Alagarsamy K, Thangaleela S, Bharathi M, Kesika P, Chaiyasut C. 2023. Composition, microbiota, mechanisms, and anti-obesity properties of rice bran. Foods 12:1300. doi:10.3390/foods12061300 36981226 PMC10048552

[52] Velez P, Espinosa-Asuar L, Figueroa M, Gasca-Pineda J, Aguirre-von-Wobeser E, Eguiarte LE, Hernandez-Monroy A, Souza V. 2018. Nutrient dependent cross-kingdom interactions: fungi and bacteria from an oligotrophic desert oasis. Front Microbiol 9:1755. doi:10.3389/fmicb.2018.01755 30131780 PMC6090137

[53] Heroven AK, Dersch P. 2014. Coregulation of host-adapted metabolism and virulence by pathogenic yersiniae. Front Cell Infect Microbiol 4:146. doi:10.3389/fcimb.2014.00146 25368845 PMC4202721

[54] Gabriel CL, Ferguson JF. 2023. Gut microbiota and microbial metabolism in early risk of cardiometabolic disease. Circ Res 132:1674–1691. doi:10.1161/CIRCRESAHA.123.322055 37289901 PMC10254080

[55] Wu J, Wei Z, Cheng P, Qian C, Xu F, Yang Y, Wang A, Chen W, Sun Z, Lu Y. 2020. Rhein modulates host purine metabolism in intestine through gut microbiota and ameliorates experimental colitis. Theranostics 10:10665–10679. doi:10.7150/thno.43528 32929373 PMC7482825

[56] Gao K, Mu C-L, Farzi A, Zhu W-Y. 2020. Tryptophan metabolism: a link between the gut microbiota and brain. Adv Nutr 11:709–723. doi:10.1093/advances/nmz127 31825083 PMC7231603

[57] Belvoncikova P, Maronek M, Gardlik R. 2022. Gut dysbiosis and fecal microbiota transplantation in autoimmune diseases. Int J Mol Sci 23:10729. doi:10.3390/ijms231810729 36142642 PMC9503867

[58] Hosseinkhani F, Heinken A, Thiele I, Lindenburg PW, Harms AC, Hankemeier T. 2021. The contribution of gut bacterial metabolites in the human immune signaling pathway of non-communicable diseases. Gut Microbes 13:1–22. doi:10.1080/19490976.2021.1882927 PMC7899087 33590776

[59] Boursier J, Mueller O, Barret M, Machado M, Fizanne L, Araujo‐Perez F, Guy CD, Seed PC, Rawls JF, David LA, Hunault G, Oberti F, Calès P, Diehl AM. 2016. The severity of nonalcoholic fatty liver disease is associated with gut dysbiosis and shift in the metabolic function of the gut microbiota. Hepatology 63:764–775. doi:10.1002/hep.28356 26600078 PMC4975935

[60] Jiang W, Gong L, Liu F, Ren Y, Mu J. 2021. Alteration of gut microbiome and correlated lipid metabolism in post-stroke depression. Front Cell Infect Microbiol 11:663967. doi:10.3389/fcimb.2021.663967 33968807 PMC8100602

[61] Ma Y, Sun Y, Sun L, Liu X, Zeng R, Lin X, Li Y. 2021. Effects of gut microbiota and fatty acid metabolism on dyslipidemia following weight-loss diets in women: results from a randomized controlled trial. Clin Nutr 40:5511–5520. doi:10.1016/j.clnu.2021.09.021 34656033

[62] Dai X, Gu Y, Guo J, Huang L, Cheng G, Peng D, Hao H. 2022. Clinical breakpoint of apramycin to swine Salmonella and its effect on ileum flora. IJMS 23:1424. doi:10.3390/ijms23031424 35163350 PMC8835974

[63] Song Y, Li F, Fischer-Tlustos AJ, Neves ALA, He Z, Steele MA, Guan LL. 2021. Metagenomic analysis revealed the individualized shift in ileal microbiome of neonatal calves in response to delaying the first colostrum feeding. J Dairy Sci 104:8783–8797. doi:10.3168/jds.2020-20068 34024606

[64] He Y, Wu S, Feng Y, Zong Z. 2022. Huaxiibacter chinensis gen. nov., sp. nov., recovered from human sputum. Int J Syst Evol Microbiol 72. doi:10.1099/ijsem.0.005484 35976100

[65] Wu W, Feng Y, Zong Z. 2019. Characterization of a strain representing a new Enterobacter species, Enterobacter chengduensis sp. nov. Antonie Van Leeuwenhoek 112:491–500. doi:10.1007/s10482-018-1180-z 30302649

[66] Lagier J-C, Dubourg G, Million M, Cadoret F, Bilen M, Fenollar F, Levasseur A, Rolain J-M, Fournier P-E, Raoult D. 2018. Culturing the human microbiota and culturomics. Nat Rev Microbiol 16:540–550. doi:10.1038/s41579-018-0041-0 29937540

[67] Schirmer M, Garner A, Vlamakis H, Xavier RJ. 2019. Microbial genes and pathways in inflammatory bowel disease. Nat Rev Microbiol 17:497–511. doi:10.1038/s41579-019-0213-6 31249397 PMC6759048

[68] Li C, Luan Z, Zhao Y, Chen J, Yang Y, Wang C, Jing Y, Qi S, Li Z, Guo H, Xu W, Zhao B, Wu C, Wang S, Yang Y, Sun G. 2022. Deep insights into the gut microbial community of extreme longevity in south Chinese centenarians by ultra-deep metagenomics and large-scale culturomics. NPJ Biofilms Microbiomes 8:28. doi:10.1038/s41522-022-00282-3 35440640 PMC9019030

